# Number of Publications on New Clinical Prediction Models: A Bibliometric Review

**DOI:** 10.2196/62710

**Published:** 2025-07-04

**Authors:** Banafsheh Arshi, Laure Wynants, Eline Rijnhart, Kelly Reeve, Laura Elizabeth Cowley, Luc J Smits

**Affiliations:** 1 Department of Epidemiology, CAPHRI Care and Public Health Research Institute, Faculty of Health, Medicine and Life Sciences, Maastricht University, Peter Debyeplein 1, P.O. Box 616, Maastricht, 6200 MD, The Netherlands, 31 433882821; 2 Institute for Risk Assessment Sciences (IRAS), Utrecht University, Utrecht, The Netherlands; 3 Department of Development and Regeneration, KU Leuven, Leuven, Belgium; 4 Leuven Unit for Health Technology Assessment Research (LUHTAR), KU Leuven, Leuven, Belgium; 5 Epidemiology, Biostatistics and Prevention Institute, University of Zürich, Zurich, Switzerland; 6 Population Data Science, Swansea University Medical School, Wales, United Kingdom

**Keywords:** prediction modeling, model development, external validation, public health, research waste, clinical prediction, clinical practice, online databases, health outcomes, publication records, publication

## Abstract

**Background:**

Concerns have been expressed about the abundance of new clinical prediction models (CPMs) proposed in the literature. However, the extent of this proliferation in prediction research remains unclear.

**Objective:**

This study aimed to estimate the total and annual number of CPM development-related publications available across all medical fields.

**Methods:**

Using a validated search strategy, we conducted a systematic search of literature for prediction model studies published in Pubmed and Embase between 1995 and the end of 2020. By taking random samples for each year, we identified eligible studies that developed a multivariable model (ie, diagnostic or prognostic) for individual-level prediction of a health outcome across all medical fields. Exclusion criteria included development of models with a single predictor, studies not involving humans, methodological studies, conference abstracts, articles with unavailable full text, and those not available in English. We estimated the total and annual number of published regression-based multivariable CPM development articles, based on the total number of publications, proportion of included articles, and the search sensitivity. Furthermore, we used an adjusted Poisson regression to extrapolate our results to the period 1950‐2024. Additionally, we estimated the number of articles that developed CPMs using techniques other than regression (eg, machine learning).

**Results:**

From a random sample of 10,660 articles published between 1995 and 2020, 109 regression-based CPM development articles were included. We estimated that 82,772 (95% CI 65,313‐100,231) CPM development articles using regression were published, with an acceleration in model development from 2010 onward. With the addition of articles that developed non-regression-based CPMs, the number increased to 147,714 (95% CI 125,201-170,226). After extrapolation to the years 1950‐2024, the number of articles increased to 156,673 and 248,431 for regression-based models and total CPMs, respectively.

**Conclusions:**

Based on a representative sample of publications from the literature, we estimated that nearly 250,000 articles reporting the development of CPMs across all medical fields were published until 2024. CPM development–related publications continue to increase in number. To prevent research waste and close the gap between research and clinical practice, focus should shift away from developing new CPMs to facilitating model validation and impact assessment of the plethora of existing CPMs. Limitations of this study include restriction of search to articles available in English and development of the validated search strategy prior to the popularity of artificial intelligence and machine learning models.

## Introduction

Concerns have been expressed about the abundance of new clinical prediction models (CPMs) proposed in the literature and their limited application in clinical practice, pointing to a high level of research waste [1,2]. Despite efforts to regulate model development and shift focus to model evaluation and implementation, new models appear to be developed at an ever increasing rate [3-5]. Although the number of published CPMs has been reported for specific medical fields or target groups [6-8], a comprehensive overview of the publication of CPM development studies across the spectrum of health care practice does not currently exist. In this study, we estimated the total and annual number of CPM development articles available across all medical fields.

## Methods

### Study design

This study is a bibliometric analysis of a previously published protocol [9], registered on the Open Science Framework Registries platform [10]. We conducted a systematic search of prediction model articles published between January 1, 1995, and December 31, 2020, in PubMed and Embase, using a validated search strategy for retrieval of clinical prediction modeling studies [sensitivity: 98.2% (91.5%‐99.9%) and specificity: 86.1% (85.4%‐86.7%)] (see Textbox 1) [11-15]. Publication records including titles and abstracts from the searched online databases were stored separately based on each publication year.

Textbox 1. **Search terms for prediction models developed and validated by Ingui and Rogers**

‘(Validat$ OR Predict$.ti. OR Rule$) OR (Predict$ AND (Outcome$ OR Risk$ OR Model$)) OR ((History OR Variable$ OR Criteria OR Scor$ OR Characteristic$ OR Finding$ OR Factor$) AND (Predict$ OR Model$ OR Decision$ OR Identif$ OR Prognos$)) OR (Decision$ AND (Model$ OR Clinical$ OR Logistic Models/)) OR (Prognostic AND (History OR Variable$ OR Criteria OR Scor$ OR Characteristic$ OR Finding$ OR Factor$ OR Model$))’

This study has been prepared in accordance with the reporting bibliometric reviews of the biomedical literature (BIBLIO) guideline [16]. The PRISMA (Preferred Reporting Items for Systematic reviews and Meta-Analyses) [17] elements have been incorporated into the description of the systematic search.

### Random Sampling

We aimed to obtain an unbiased stratified sample of at least 100 published CPM articles. Based on published search string metrics, positive predictive value 3.5% (95% CI 2.7%‐4.6%), we calculated that 2860 records needed screening; therefore, 110 records were randomly selected per publication year. As the target sample size of 100 articles—with a minimum of one development article per year— was not reached after the screening process, we increased the sample size in increments of 30 articles per year until at least 100 articles were included. Details about the sample size calculation and random selection of records are provided in Multimedia Appendix 1 and the published protocol [9].

### Eligibility, Screening Process and Data Extraction

Articles were eligible for inclusion in this study if they described the development or external validation of a multivariable model [18]. Studies that described development of a regression-based multivariable prediction model for a health outcome were the focus of the current analysis. Studies that reported development of a prediction model without using logistic, proportional hazards, or linear regression were designated as non-regression–based development articles (eg, machine learning models or scoring rules based on multiple unadjusted bivariate associations). Studies updating existing models were also categorized as development articles.

Articles that externally validated a CPM in a different population or in a different time period were designated as validation articles. Of note, an article could be of more than one type (eg, CPM development and external validation article). A random train-test split was not considered external validation. Finally, decision curve analysis (net benefit) was regarded as a method of validation.

We excluded articles that did not present any developed prediction models or external validation in the main results sections (eg, etiologic studies, risk factor assessments, reviews). Articles were also excluded if they were based on a single predictor; reported development or validation of a diagnostic questionnaire (eg, for diagnosis of depression); presented models that were neither diagnostic nor prognostic; did not yield individual-level predictions; did not involve humans; or if they were methodological studies. Articles that were not available in English; conference abstracts; and those without available full texts were also excluded.

Six reviewers (BA, ER, KR, LC, LS, and LW) independently screened titles and abstracts, in pairs. After selection of potential eligible articles, two independent researchers (BA and ER) reviewed the full text articles for eligibility and extracted data. Disagreements were discussed among the reviewers and unresolved conflicts were solved during team consensus meetings. Extracted data included medical domain (based on outcome or target population, or both); publishing journal; publication year; country; study design; study setting; data sources; study population; sample size; outcomes; number of models; model type (diagnostic or prognostic); internal or external validation; performance measures (eg, discrimination, calibration); and model presentation (model equation, nomogram, sum score, etc.).

### Data Analysis

Characteristics of eligible regression-based CPM development articles were described using frequencies (n) and percentages (%) for categorical data. We categorized articles by medical field, geographical origin, model type (diagnostic or prognostic), and other characteristics. Next, we estimated the annual number of CPM development articles using the following formula:


$$N_{CPM\;development\;articles}=\left(\frac{N_{identified\;CPM\;development\;articles}}{N_{screened\;articles}}\right)\times N_{search\;hits}\times \frac{1}{search\;sensitivity}$$

To estimate the overall number of articles, we used the weighted average of year-specific proportions of CPM development articles, weighted by the number of hits per year using a conventional estimator of proportion based on a stratified sample [19]. Further details, including calculation of 95% CI is shown in Multimedia Appendix 1. As the search string was developed for identification of regression-based CPMs, we focused primarily on these models for our primary analysis. Additionally, we extrapolated our estimates to 1950‐2024 using Poisson regression adjusted for publication year. We compared the fit of models with a quadratic term and a linear term for publication year, which showed no apparent difference between the two models (*P=*.82). Therefore, a linear term was used for the publication year.

In additional analyses, we estimated the total and annual number of articles that reported development of non-regression–based CPMs and external validation articles using the above-mentioned formula, and calculated their respective 95% CIs. For sensitivity analyses, we recalculated our estimations using search sensitivity reported in a validation study by Geersing et al [15].

### Ethical Considerations

This study was deemed as conforming to the ethics standards of Maastricht University by the ethics committee (case number: FHML-REC/2023/066).

## Results

**Figure 1.**
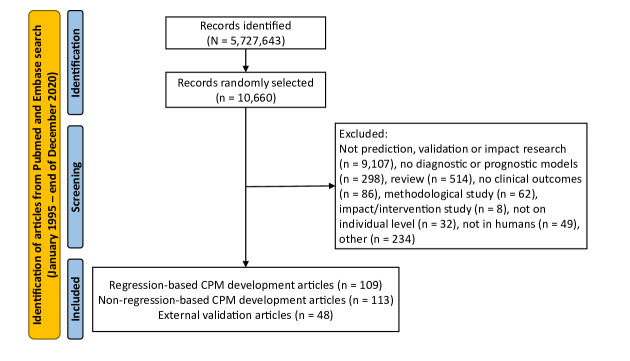
PRISMA chart for screening and selection of eligible articles. CPM: clinical prediction model.

Our search yielded 5,727,643 publications, from which a random sample of 10,660 (410 articles per year) were screened. A total of 222 CPM development articles were identified. including 109 reporting on regression-based models and 113 on non-regression–based models (Figure 1). Characteristics of the regression-based articles are shown in Table 1. Most articles were from North America 41 (37.6%), Europe 37 (33.9%), and Asia 25 (22.9%). The most common medical fields for models development were cardiovascular medicine 22 (20.1%), gynecology and obstetrics 16 (14.7%), and gastroenterology 14 (12.8%). Across all medical fields, 38 (34.9%) of the articles were related to oncology (Table S1 in Multimedia Appendix 1). In addition, 67 (61.5%) of articles described prognostic models while 42 (38.5%) described diagnostic models. Model calibration and discrimination were reported in 31 (28%) and 87 (80%) of the articles, respectively; only 37 (33.9%) of the articles presented their final model.

**Table 1.** Characteristics of included regression-based CPM development articles.

Study characteristics	Articles (N=109), n (%)
Continent	
North America	41 (37.6)
Europe	37 (33.9)
Asia	25 (22.9)
South America	1 (0.9)
Australia	1 (0.9)
Africa	1 (0.9)
North America-Europe	3 (2.8)
Medical field	
Cardiovascular disease	22 (20.1)
Gynecology and Obstetrics	16 (14.7)
Gastroenterology	14 (12.8)
Neurology	13 (11.9)
Urology	8 (7.3)
Pulmonology	7 (6.4)
Pediatrics	6 (5.5)
Psychiatry	5 (4.6)
Other fields^a^	18 (16.5)
Type of model	
Prognostic	67 (61.5)
Diagnostic	42 (38.5)
Calibration information given	31 (28.0)
Calibration plot reported	15 (13.8)
Discriminative performance reported	87 (80.0)
C-statistic reported	65 (59.6)
Validation of developed models	
Internal validation	27 (24.8)
External validation	14 (12.8)
Internal validation technique	
Random split of dataset into development and testing sets	8 (7.3)
Cross-validation	7 (6.4)
Bootstrapping	9 (8.3)
Temporal split of dataset into development and testing sets	1 (0.9)
Unclear	3 (2.8)
Final model presented	37 (33.9)

a Other fields: Infectious disease: 4 (3.7%); Endocrinology: 3 (2.7%); Hematology: 3 (2.7%); Dermatology: 2 (1.8%); Critical care: 2 (1.8%); Orthopedic surgery: 2 (1.8); Ophthalmology: 1 (0.9%); Emergency medicine: 1 (0.9%).

**Figure 2.**
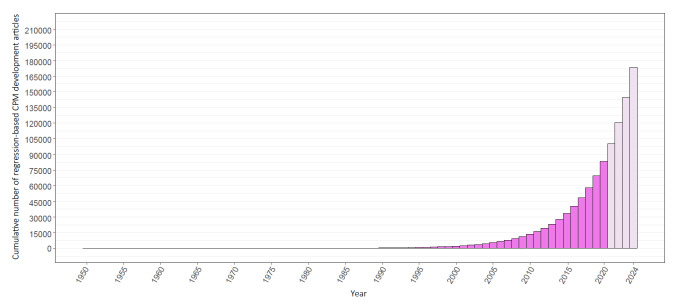
Cumulative estimated number of regression-based CPM development articles published between 1950 and 2024.

**Figure 3.**
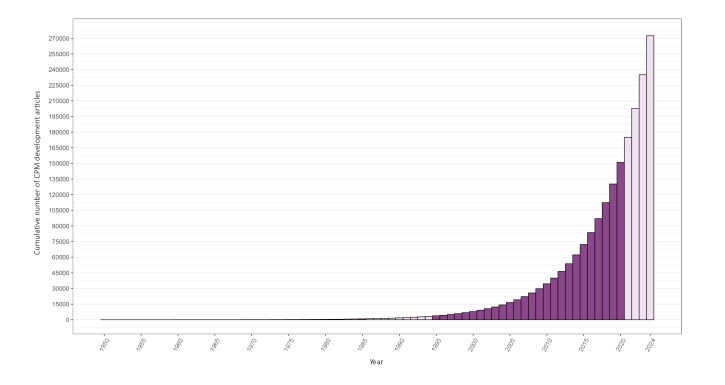
Cumulative estimated number of regression and non-regression–based CPM development articles between 1950 and 2024.

The estimated number of published regression-based CPM development articles from 1995 to 2020 was 82,772 (95% CI 65,313‐100,231), with the number of these articles rapidly increasing from 2010 onward (Figure 2, darker area). The number of regression-based CPM development articles that were published increased to 156,673 after extrapolating the findings to 1950‐2024 (Figure 2, lighter area). Furthermore, an estimated 64,942 (95% CI 50,484‐79,400) non-regression–based development articles were published between 1995 and 2020 (Figure 3, darker area), increasing the estimated total number of CPM development articles during this period to 147,714 (95% CI 125,201‐170,226). When expanded to 1950‐2024, this number increased to 248,431 (Figure 3, lighter area).

In additional analysis**,** the estimated number of CPM external validation articles (n=48) published between 1995 and 2020 was 36,394 (95% CI 24,837‐47,952) (Figure S1 in Multimedia Appendix 1). The estimated number of articles based on search sensitivity reported by Geersing et al [15] are presented in Multimedia Appendix 1.

## Discussion

### Summary

More than 150,000 articles reporting new regression-based CPMs have been published in the medical literature up to 2024, with the number increasing rapidly in recent years—about 10,000 per year. After the addition of non-regression models, the current total approaches 250,000 articles.

Trends in the numbers of model development studies had not been systematically assessed across different medical fields to date. Previously, CPM development for cardiovascular outcomes showed a three-fold increase between 2005-2014, compared to 1995‐2004 [5]. Further, a sharp increase was observed in the number of models developed using electronic health record data between 2009‐2019 [3]. Our study shows that despite all regulatory efforts, the growth in model development is ever increasing and is not limited to a specific field [11]. This surge in model development publication has been attributed to the growing accessibility to data and analytical tools [12,20]. However, the excessive model development may obscure decent models and hinder their evaluation [2]. Without further evaluation such as external validation, these models are unlikely to be fit for adoption in clinical practice.

The proportion of CPMs that undergo external validation remains the topic of ongoing research [9,21]. Among 127 selected CPMs developed before 2010, the probability of external validation within 5 years was only 16% [21]. At such rates, the current surge in model development is bound to produce excess research waste. Adequate methodology and transparent reporting may increase the chances of validation [5,22]. In our study, two-thirds of the development articles did not report the final model. With such an omission, the likelihood that a model will be further evaluated is limited.

Our study benefits from a long time span across more than two decades, assessment of a random sample, and a validated search strategy. However, certain limitations should be acknowledged. Our search was restricted to articles available in English. In addition, the validated strategy that we used was developed prior to the rise in popularity of machine learning and artificial intelligence models. Therefore, our estimates may be considered a lower bound of the actual abundance of CPM development. Despite this, our findings underscore the implications for the current enthusiasm and rapid growth of machine learning and artificial intelligence models. Given the emphasis on retraining, we reiterate that these models also need to undergo rigorous evaluation [7,23,24]. The lack of interpretability of these complex models poses additional challenges; when it is difficult to understand how a model arrives at its predictions, rigorous validation is all the more important.

### Implication for Researchers and Stakeholders

To enhance efficiency, prediction research must shift from developing new CPMs to evaluating existing ones [1]. Targeted validation and impact assessment of current CPMs, along with active dissemination and implementation of prediction model-based innovations are crucial steps in minimizing research waste and bridging the gap between research and clinical practice [25]. The responsibility for advancing model evaluation beyond development falls on researchers, clinicians, funding agencies, and other stakeholders. Future validation efforts may depend on both pre-development factors (eg, a clearly defined clinical need, clinician, or public demand) and post-development considerations (eg, model complexity, feasibility of use or the absence of a dissemination or implementation plan) [5]. Additionally, the emphasis on novelty by funding agencies may inadvertently discourage evaluation of existing models. We also advocate for greater transparency in reporting (including presentation of model equations), alongside adherence to contemporary methodological standards by researchers.

### Conclusion

Based on a random sample of publications from a validated search strategy, we estimate that nearly 250,000 articles reporting the development of clinical prediction models were published in the literature until 2024. The proliferation of model development, regardless of medical field has accelerated at an unprecedented rate in recent years. It is critical to shift focus from model development to model validation and assessing impact on health care.

## Supplementary material

10.2196/62710 Multimedia Appendix 1 Description of methods and data analysis.

10.2196/62710 Checklist 1 PRISMA (Preferred Reporting Items for Systematic reviews and Meta-Analyses) checklist.

## Abbreviations

CPM: clinical prediction model
