# Supplementary material for: Number of Publications on New Clinical Prediction Models: A Bibliometric Review
Source: JMIR Med Inform. 2025 Jul 4;13:e62710. doi: 10.2196/62710 (PMC12252138; doi:10.2196/62710)
Supplement: Multimedia Appendix 1 [file medinform-v13-e62710-s001.docx]

**Supplemental description of methods**

This study is an secondary analysis to a larger systematic review for which the protocol has been previously published[1] and registered in the Open Science Framework (OSF) Registries platform.[2] The methods section for this study has been prepared in accordance with the Preferred reporting items for systematic reviews and meta-analyses (PRISMA) statement[3].

**Sample size**

We used the Ingui search string. Based on its estimated positive predictive value (PPV (95%CI) 3.5% (2.7-4.6), we expected needing to screen 110 randomly selected records from the search hits per year, 2860 in total, to identify at least 100 eligible articles in each year. Details about the random selection of abstracts are provided elsewhere (2). The unselected abstracts of each publication year were held in reserve. As the target sample size of 100 articles developing a CPM using regression, and one minimum development article per year, was not reached after the screening process, we increased the sample size by increments of 30 articles per year, until we had included at least 100 CPM development articles. The target sample size of 100 development articles was set for the second stage of the project, where we must be able to estimate the proportions of developed CPMs that are later validated, assessed for impact, implemented and used with reasonable precision. With this sample size we will reach Wilson score CI half-width (i.e., margin of error) of 2.6% assuming a proportion of 1% and score CI half-width of 8% assuming a proportion of 20%.

**Data analysis**

Characteristics and reporting of the eligible regression-based CPM development articles were described using Numbers (N) and percentages (%) for categorical data. Next, we estimated the number of developed CPM development articles from 1995 until the end of December 2020:

N_publications each year_= P_year_ * N_year_ / 0.982

Where N_year_ is the number of hits for each year, P_year_ is the number of identified development publications for each year divided by the number of randomly screened abstracts (410), and 0.982 is the previously reported sensitivity of the search string.[4]

To estimate the overall number of articles, we first estimated P_overall_ by using the weighted average of stratum-specific P_year_, weighted by the number of hits per stratum (N_year_), using a conventional estimator of a proportion based on a stratified sample[5]:

P_overall_=1/N_total_* $\sum_{n=1995}^{2020}$P_year_ * N_year_

Overall estimated number of articles= P_overall_* N_total_ / 0.982

Where N_total_ is the overall number of hits (5,344,272) and 0.982 is again the reported sensitivity of the search string. Next, we calculated stratum-specific and total variance to use in our estimate of the 95%CI around the estimated number of articles.[4]

Estimated variance per year (Var_year_):

Var_year_ = (N_year_ - 410)/ N_year_ * P_year_ *(1- P_year_)/(410-1)

Estimated variance of the total stratified sample:

Var_total_ = 1/ N_total_^2^ *$\sum_{n=1995}^{2020}$(Nyear)^2^ * Var_year_

Calculating 95%CI, taking sensitivity into account:

(Overall proportion of articles ± 1.96* √ Var_total_)* N_total_/0.982

In sensitivity analysis, to account for the years missed before 1995, we extrapolated our results by fitting a generalized linear model with a Poisson link function with year as the only independent variable on our estimations and the number of articles for years 1995-2020 as outcomes. We then estimated number of articles for years 1950-1995 and 2021-2024 based on the resulting model. The earliest popular example of an evidence-based risk stratification model in medicine[6], the invention of ROC methodology[7], and the popularization of logistic regression[8] can all be situated in the early 1950s. Nonetheless, some linear regression CPMs might have been published before the 1950s, but we assume the numbers are negligible before this time.

Additionally, we quantified the number (95%) of non-regression based CPMs during the study period using the same method, even though the search string is not optimal to identify machine learning publications and may yield an underestimation. Subsequently, estimation of the number of CPM validation articles was performed in the same manner.

**Results based on additional validation metrics**

After incorporating the sensitivity of the search strategy validated by Geersing et al[9] (sensitivity: 0.78), the estimated number of regression-based CPM development articles from 1995 to 2020 was 104,208 (82,227-126,188). And, the estimated number, including non-regression-based development articles, was 185,968 (157,625-214,311). Finally, the number of external validation articles in this period increased to 45,820 (95% CI 31,269-60,371) based on this sensitivity.

| Medical field | *Oncology related articles, n*(%)^a^ |
| --- | --- |
| Cardiovascular disease | - |
| Gynecology and Obstetrics | 10 (9.2) |
| Gastroenterology | 9 (8.3) |
| Neurology | - |
| Urology | 6 (5.5) |
| Pulmonology | 5 (4.6) |
| Pediatrics | 1 (0.9) |
| Psychiatry | - |
| Critical care | 1 (0.9) |
| Infectious disease | - |
| Hematology | 3 (2.8) |
| Orthopedics | - |
| Endocrinology | 3 (2.8) |
| Dermatology | - |
| Ophthalmology | - |

**Table S1.** Number (%) of included regression-based CPM development articles from each field in oncologic settings or for oncology outcomes

**^a^** Percentages are reported out of the total number of regression-based CPM development articles

**Figure S1.** Cumulative estimated number of external validation articles between 1950 and 2020


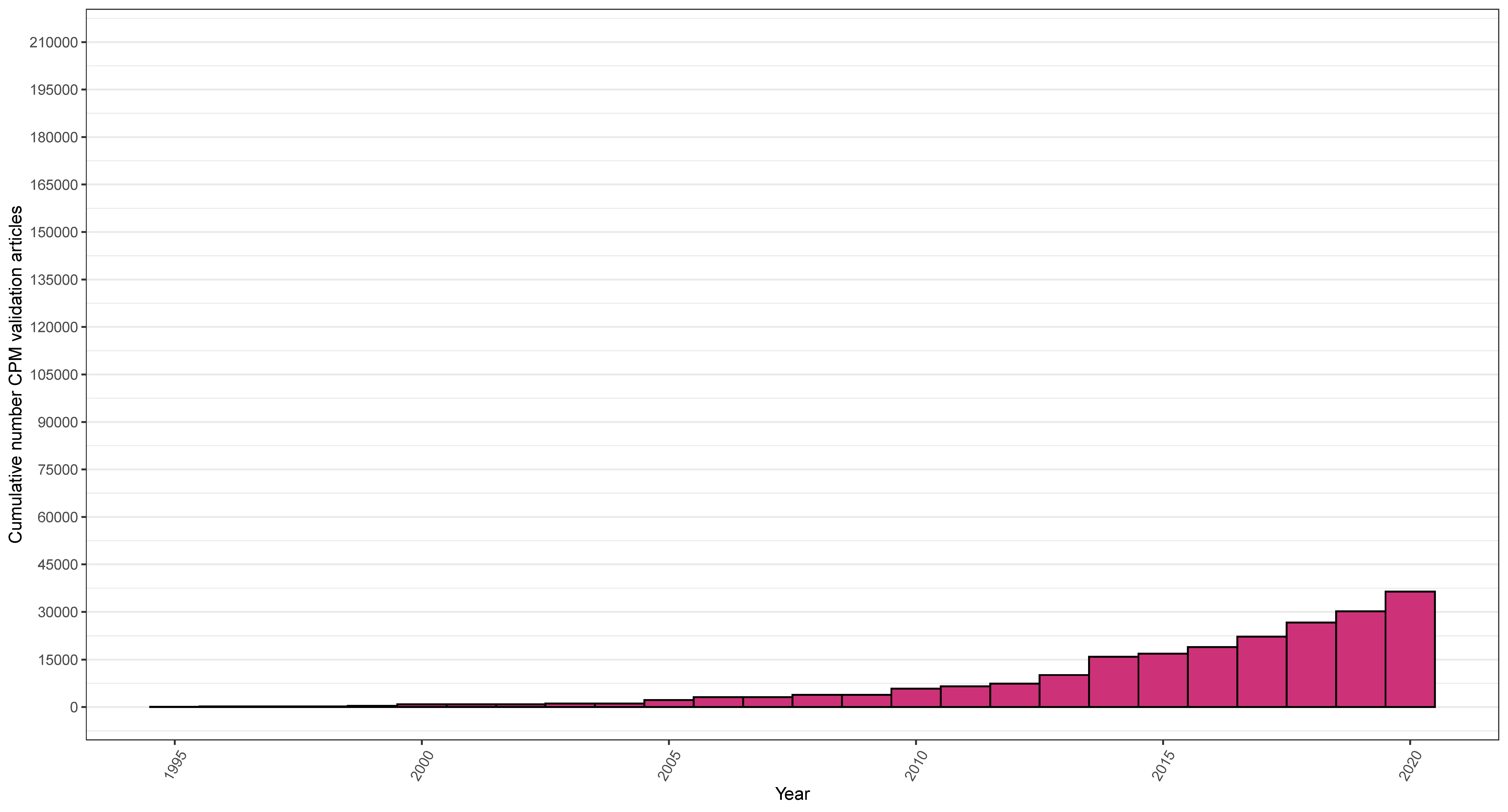


Figure shows the estimated number of CPM external validation articles published between 1950 and 2020.

**References**

1. Arshi B, Wynants L, Rijnhart E, Reeve K, Cowley LE, Smits LJ. What proportion of clinical prediction models make it to clinical practice? Protocol for a two-track follow-up study of prediction model development publications. BMJ open. 2023;13(5):e073174.

2. Arshi B, Wynants L, Smits LJ, Cowley LE, Reeve K, Rijnhart E. What proportion of clinical prediction models make it to clinical practice? Protocol for a two-track follow-up study. OSF. 2023; available at: osf.io/nj8s9.

3. Page MJ, McKenzie JE, Bossuyt PM, Boutron I, Hoffmann TC, Mulrow CD, et al. The PRISMA 2020 statement: an updated guideline for reporting systematic reviews. Systematic reviews. 2021;10(1):1-11.

4. Ingui BJ, Rogers MAM. Searching for clinical prediction rules in MEDLINE. Journal of the American Medical Informatics Association. 2001;8(4):391-7.

5. Lohr SL. Sampling: design and analysis: CRC press; 2021. ISBN: 1000478238.

6. Apgar V. A proposal for a new method of evaluation of the newborn. Classic Papers in Critical Care. 1952;32(449):97.

7. Peterson WW, Birdsall TG. The theory of signal detectability: Part I, the general theory: Part II, applications with Gaussian noise. 1953.

8. Cramer JS. The origins of logistic regression. 2002.

9. Geersing G-J, Bouwmeester W, Zuithoff P, Spijker R, Leeflang M, Moons K. Search filters for finding prognostic and diagnostic prediction studies in Medline to enhance systematic reviews. PloS one. 2012;7(2):e32844.
